# New *Cydia pomonella* granulovirus strain with high entomopathogenic activity

**DOI:** 10.1128/mra.00538-24

**Published:** 2024-08-20

**Authors:** Aleksandra A. Tsygichko, Anzhela M. Asaturova, Gennady V. Vasiliev, Alexandra I. Klimenko, Sergey A. Lashin, Tatiana N. Lakhova

**Affiliations:** 1 Federal State Budgetary Scientific Institution, Federal Research Center of Biological Plant Protection, Krasnodar, Russia; 2 Federal Research Center, Institute of Cytology and Genetics SB RAS, Novosibirsk, Russia; DOE Joint Genome Institute, Berkeley, California, USA

**Keywords:** CpGV, *Cydia pomonella* granulovirus, insect baculovirus, bioinsecticide

## Abstract

We report a genome of CpGV from the bioresource collection of the Federal Research Center of Biological Plant Protection “State Collection of Entomoacariphages and Microorganisms.” Its sequence is 123,862 bp. The genome under study demonstrates a degree of similarity of more than 99% with reference NC_002816 from the NCBI RefSeq database.

## ANNOUNCEMENT

One of the safe, effective, and narrowly focused bioinsecticides against *Cydia pomonella* is CpGV-based products (1). CpGV belongs to the class *Naldaviricetes*, order *Lefavirales*, family *Baculoviridae*, genus *Betabaculovirus*, and species *Betabaculovirus cypomonellae*. Such preparations are widely used, but with their long-term application, insects develop resistance, which can be overcome by using new strains as a basis (2, 3). We present two partial genome sequences of a previously unknown CpGV strain from the bioresource collection of the Federal Research Center of Biological Plant Protection “State Collection of Entomoacariphages and Microorganisms.”

The strain was isolated in Krasnodar, Russia, in 2010. During the identification process, total viral DNA was isolated from the biomaterial of infected *C. pomonella* caterpillars by centrifuge sedimentation using the PureLink Genomic DNA Mini Kit (Invitrogen). DNA concentration was determined fluorimetrically on a Qubit device using the High Sensitivity kit. The KAPA DNA Prep kit was used to develop a genomic library. For barcoding, UDI77 index from the KAPA Unique Dual-Indexed Adapter Kit was used. The amplification of the library was carried out during nine cycles of PCR; the library was purified by adding an equal volume of Agencourt AMPure XP. The quality of the library was determined using a BA2100 bioanalyzer. Molarity was calculated using data from a bioanalyzer and Qubit 2.0 fluorimeter; the final library was normalized to 4 nM. Sequencing was carried out on a NextSeq550 device with 2 × 150 bp paired-end reads according to the manufacturer’s protocol producing 27.2 mln pair-end reads. To collect and analyze the data, the programs FastQC, Fastp, Samtools, Spades, MinYS, Pilon, Gfinisher, Prokka, and Quast (4–11) were used (12). The average depth of coverage of the novel genome in the read library 693,08x. NC_002816, obtained from the NCBI RefSeq database, was used as the reference genome. The degree of similarity between the studied coding-complete genome and the reference was determined using the average nucleotide identity parameter, which was calculated using the FastANI program and gplots (13).

The strain was designated BZR GV L-3. The coding-complete genome of the BZR GV L-3 strain had a length of 123,862 base pairs and contained 132 coding sequence (CDS), which were mostly similar to the genes of other baculoviruses. The G+C nucleotide distribution was 45%. A comparison of the coding-complete genome of the BZR GV L-3 baculovirus strain with the sequence of the reference genome NC_002816 allowed us to determine a similarity of 99.4%. It was found that the studied CpGV genome contained genes encoding the “insecticidal” proteins IAP, cathepsin, MMP, and chitinase, which allows it to be a promising agent for the development of bioinsecticides based on it (14).

## Data Availability

Partial genome sequences of the BZR GV L-3 strain (CpGV) obtained during the study have been deposited in GenBank under accession numbers OR651295 and OR651296. The initial NGS data are presented in the SRA NCBI.
